# Temporal Effects of Preservation on the Shape and Size of Yellow Perch: Implications for Morphological Analyses

**DOI:** 10.1002/ece3.71968

**Published:** 2025-09-25

**Authors:** Tyler J. Hoyt, Carl R. Ruetz

**Affiliations:** ^1^ Annis Water Resources Institute Grand Valley State University Muskegon Michigan USA

**Keywords:** fish preservation, geometric morphometrics, morphology, yellow perch

## Abstract

Long‐term preservation of fishes often involves fixation in formalin followed by preservation in ethanol. This preservation technique can alter the shape and size of specimens, although the effects are often species and stage specific. In this study, we evaluated the temporal effects of preservation on the shape and body size of adult Yellow Perch ( *Perca flavescens* ). We sampled 42 Yellow Perch from three lakes directly connected to Lake Michigan, USA. Upon capture, fish were transported to the laboratory, fixed in formalin, and then preserved in ethanol. Measurements (length, mass, and photograph to analyze shape) were made on fresh fish (i.e., prior to preservation) and 21, 47, and 61 days after being preserved in ethanol. Shape was analyzed using geometric morphometrics. We identified significant changes in shape, and to a lesser degree, size. The most significant shape change occurred in the comparison of pre‐preserved versus 21 days, with most of this variation attributed to dorsal bending. Shape changed slightly when comparing 21‐ versus 47‐day but was not detectable between 47‐ and 61‐days post preservation. Total length and to a lesser degree centroid size and mass also differed over time, but total length and centroid size did not follow a similar pattern to shape. Changes in total length and mass were minor (< 1%) and were observed when comparing 47 vs. 61 days and pre‐preserved vs. 21 days, respectively. Changes in centroid size were modest (~5%) and were observed paradoxically when comparing 47 vs. 61 days (similar to total length). Although most of the observed differences in the shape and size of adult Yellow Perch were slight, we recommend the conservative approach of only comparing fresh individuals with other fresh individuals and preserved individuals with other individuals preserved for the same amount of time when performing analyses of shape and size.

## Graphical abstract

We assessed the shape and size of adult Yellow Perch when fresh fish (i.e., prior to preservation) and 21, 47, and 61 days after being preserved in ethanol. Shape, centroid size, total length, and mass were influenced by preservation. Although most of the observed differences in the shape and size of adult Yellow Perch were slight, we recommend the conservative approach of only comparing fresh individuals with other fresh individuals and preserved individuals with other individuals preserved for the same amount of time when performing analyses of shape and size.

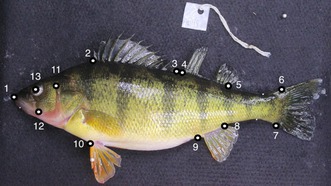

## 1 Introduction

Analysis of the size and shape of fishes is an important part of describing a species' natural history. Preserved specimens are commonly used in a variety of studies that use shape or size to answer ecological and evolutionary questions. Moreover, preserved specimens have long been housed in museum collections to provide an invaluable record of biodiversity (Jennings et al. 2012). Morphological studies using preserved specimens range from defining species (Johnson et al. 2004) and understanding the diversity among fish populations (Taylor et al. 2006) to assessing length–weight relationships (Jellyman et al. 2013). The use of preserved specimens is often necessary when fish cannot be processed the same day as collection due to logistical constraints or when taking advantage of museum collections. Common preservation techniques include storage on wet ice (Kocovsky and Knight 2012), dry ice (Tardif et al. 2005), formalin (Taylor et al. 2006), ethanol (April et al. 2013), or through a combination of these methods. Any analysis of preserved specimens makes at least two assumptions: (1) specimens retain their natural biological characteristics despite preservation‐induced changes in size and shape, and (2) any biological differences among groups are of greater magnitude than differences caused by preservation and other forms of measurement error (i.e., difference between measured quantity and true value). Therefore, understanding the effects of preservation on the shape and size of fishes is crucial to draw valid conclusions from preserved specimens.

Formalin fixation and ethanol preservation is widely used for preserving fish (Hubbs and Lagler 2004; Jennings et al. 2012). Nevertheless, there are substantial nuances associated with fish preservation that should be acknowledged when evaluating effects on size and shape. For instance, the duration between euthanasia and placement in preservative, the use of incision in the abdomen or injections to facilitate penetration of preservatives, and the exact concentrations and exposure durations of preservatives can vary among researchers (Hubbs and Lagler 2004; Jennings et al. 2012), all of which may affect shape. Regardless of the method of preservation, researchers have long recognized that preservation can alter the shape and size of the specimens (Hjörleifsson and Klein‐MacPhee 1992; Mabee et al. 1998; Paradis et al. 2007; Kočovský 2016; Lee et al. 2012; Fruciano et al. 2020; Sotola et al. 2019; Lorencen et al. 2021). Early studies using linear measurements revealed that fish commonly shrink due to formalin fixation and ethanol preservation (Leslie and Moore 1986; Shields and Carlson 1996). More recently, researchers have used geometric morphometrics to better understand the shape changes induced by preservation (Berbel‐Filho et al. 2013; Gaston et al. 2013; Fruciano et al. 2020; Sotola et al. 2019; Lorencen et al. 2021). This method uses landmark coordinates to capture and analyze shape variation. Unlike traditional morphometric approaches, which rely on linear measurements, angles, or ratios, geometric morphometrics retains the spatial relationship between landmarks, allowing for a more detailed, size‐independent analysis of shape (Zelditch et al. 2012). Preservation‐induced shape changes can be influenced by several factors, including the method of preservation (Fruciano et al. 2020), the amount of time specimens are preserved (Sotola et al. 2019; Lorencen et al. 2021), and the species, of fish that is being preserved (Sotola et al. 2019).

The Yellow Perch ( *Perca flavescens* ) is an ecologically and economically important fish in North America (Scott and Crossman 1973; Becker 1983). Complex patterns of habitat use and spatial population structure in the Laurentian Great Lakes of North America (Schoen et al. 2016; Chorak et al. 2019; Senegal et al. 2020) make the morphology of Yellow Perch of ecological and evolutionary interest (e.g., Parker et al. 2009; Kočovský et al. 2013; Yin et al. 2025). The effect of ethanol preservation on adult Yellow Perch is largely unknown, with studies assessing preservation‐induced changes on the size of Yellow Perch limited to larval and juvenile forms (Fisher et al. 1998; Paradis et al. 2007). Studies assessing adult Yellow Perch used formalin instead of ethanol as a preservative (Engel 1974; Stobo 1972). The changes observed in these studies were influenced by the method of preservation and the size of the fish. Larval and juvenile Yellow Perch preserved in ethanol were found to shrink in both length and weight (Paradis et al. 2007). Adult Yellow Perch preserved in formalin were found to shrink in length and increase in weight (Engel 1974; Stobo 1972), while juvenile Yellow Perch decreased in both length and weight (Paradis et al. 2007). To date, studies have not assessed the effects of ethanol preservation on adult Yellow Perch, nor have they assessed preservation‐induced changes in shape. The Yellow Perch differs in both size and morphology throughout ontogeny (Graeb et al. 2005, 2006), so a reasonable assumption is that preservation may affect adult Yellow Perch differently (Smith and Walker 2003). Additionally, questions remain about how time influences the effects of preservation.

Our overall goal was to assess the temporal effects of a commonly used preservation technique on the shape and size of adult Yellow Perch. Specifically, we used a combination of linear measurements and geometric morphometrics to test two hypotheses: (i) formalin fixation and ethanol preservation lead to a decrease in the length and mass of adult Yellow Perch over time (Paradis et al. 2007) and (ii) formalin fixation and ethanol preservation cause changes in shape such as the expansion of the abdominal and caudal regions over time (Sotola et al. 2019).

## 2 Methods

We collected Yellow Perch using 7.62‐cm stretch‐mesh gill nets in White Lake (43.37559°N, 86.38834°W), Arcadia Lake (44.48342°N, 86.23897°W), and Lake Macatawa (42.77867°N, 86.18477°W), which are drowned river mouth lakes located on the eastern shore of Lake Michigan, part of the Laurentian Great Lakes (Mader et al. 2023). Drowned river mouth lakes are lake‐like habitats that connect tributaries to Lake Michigan (Mader et al. 2023), and Yellow Perch commonly occur in these habitats (Janetski et al. 2013; Janetski and Ruetz 2015). We kept 14 Yellow Perch that were > 200 mm total length (TL) from each lake (*n* = 42). The sampling protocol was reviewed and approved by Grand Valley State University's Institutional Animal Care and Use Committee (protocol #22‐11‐A). Fish were captured during November–December 2022, euthanized with tricaine methanesulfonate (MS‐222) on the day of capture, and immediately transported to the laboratory on wet ice. The Yellow Perch included in this study were likely seasonal migrants from Lake Michigan. Yellow Perch from Lake Michigan are known to use drowned river mouth lakes during the winter and are often captured in profundal habitats that were sampled for this study (Chorak et al. 2019; Senegal et al. 2020). Yellow Perch in the main basin of Lake Michigan are not genetically distinct (Schraidt et al. 2023; Yin et al. 2025), which includes Lake Michigan migrants (Chorak et al. 2019). Thus, even though we sampled Yellow Perch over a large (i.e., ~190 km of Lake Michigan shoreline) geographic area, the Yellow Perch used for this study are likely the same genetic subpopulation, which is distinct from resident fish in drowned river mouths (Chorak et al. 2019; Senegal et al. 2020).

In the laboratory on the day of collection, we measured total length (mm) and mass (g), photographed each specimen in its pre‐preservation state, and uniquely marked each fish with a paper tag secured with string threaded through the mouth and operculum. We photographed the left lateral side of each specimen with fins pinned out next to a standard metric ruler on a foam board covered in black felt using a Canon Rebel t6 18 MP camera at a 39 mm focal length (Tv:1/500, Av:5.0, ISO 1600). We then fixed the specimen with 10% formalin. After 6 days in formalin, we placed the specimen in water for 1 day, and then preserved specimens in 70% non‐denatured ethanol. The specimens were preserved together in six‐gallon buckets. Special care was taken to place them vertically (nose down) in the buckets to reduce the chance of bending (i.e., if placed horizontally, specimens of greater total lengths may bend as they would reach both sides of the bucket).

We photographed and measured total length and mass of each specimen at 21, 47, and 61 days after being preserved in ethanol. Specimens were manually straightened (dorso‐ventrally and laterally) by holding the specimen straight while pinning the fish down. Ensuring that the specimens were as straight as possible when they were photographed helped to elucidate physical changes in body shape. Molding clay was used to position the specimen flat against the board.

**FIGURE 1:**
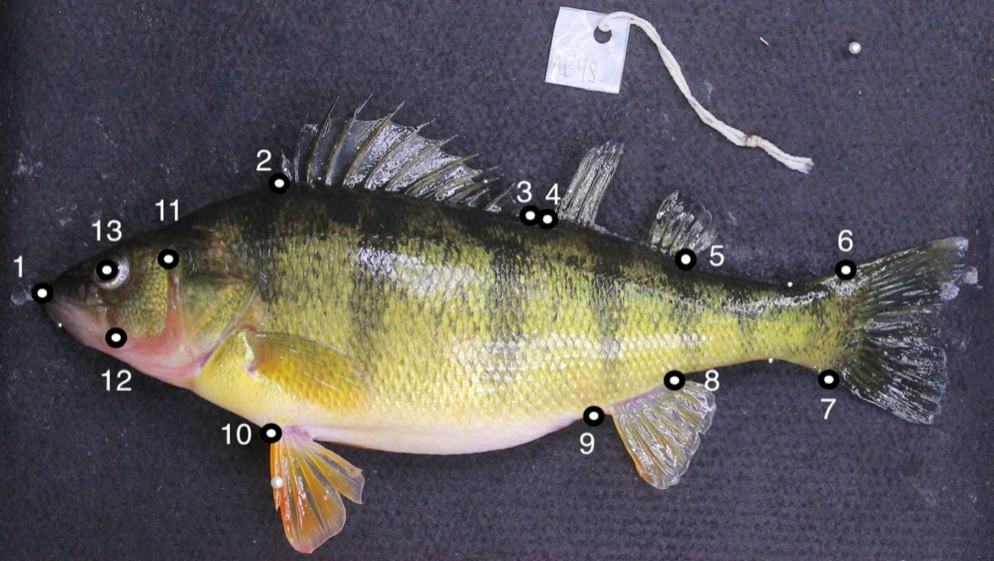
Landmarks: (1) tip of the snout, (2) anterior dorsal fin anterior insertion, (3) anterior dorsal fin posterior insertion, (4) posterior dorsal fin anterior insertion, (5) posterior dorsal fin posterior insertion, (6) caudal fin dorsal insertion, (7) caudal fin ventral insertion, (8) anal fin posterior insertion, (9) anal fin anterior insertion, (10) pectoral fin anterior insertion, (11) pre‐operculum dorsal insertion, (12) pre‐operculum proximal insertion, and (13) center of the eye.

We applied 13 digital landmarks (Figure 1) to the photograph of each specimen at four time periods (pre‐preserved, 21, 47, and 61 days) using the *geomorph* package in R (Adams and Otárola‐Castillo 2013). We superimposed the landmark configurations with a single generalized Procrustes analysis (GPA) using the “gpagen” function (Gower 1975). In addition to superimposition, this function calculates centroid size. Centroid size is a measurement of overall body size that is calculated by taking the square root of the sum‐squared distance from each landmark to the centroid. We then calculated the size‐adjusted residuals using the “procD.lm” function by regressing the Procrustes aligned coordinates against the log‐transformed centroid size (Klingenberg 2016). This removes allometry and allows for size‐independent shape analyses (Sidlauskas et al. 2011). Last, we used the size‐adjusted residuals in a principal component analysis (PCA) to reduce the dimensionality of the data.

To examine variations in size‐independent shape between different time periods, we used Hotelling's *T* ^2^ on all principal component axes, which was performed using the “repeated_measures_test” function in the *GeometricMorphometricMix* package in R (Fruciano 2018). We compared mean body shape of pre‐preserved versus 21 days, 21 versus 47 days, 47 versus 61 days, and pre‐preserved versus 61 days.

To test for differences in size between individual time periods, we conducted paired *t*‐tests using the “t.test” function in the *stats* package in R to assess differences in centroid size, total length, and mass. Based on the results of a Shapiro–Wilks normality test for each of the three variables, we found a natural‐logarithm transformation of mass was necessary to meet the assumption of normality for the paired *t*‐test; whereas no transformations were needed for total length or centroid size. We compared mean size of pre‐preserved vs. 21 days, 21 versus 47 days, 47 versus 61 days, and pre‐preserved versus 61 days.

We recognized that performing multiple pairwise comparisons of the shape and size response variables likely inflated Type I error (i.e., *α* is the probability of rejecting the null hypothesis when it is true); so we adjusted *p*‐values with the sequential Bonferroni method (Peres‐Neto 1999). However, even the sequential Bonferroni method can inflate Type II error (i.e., not rejecting the null hypothesis when it's false), which in the context of our study is arguably a more costly error (i.e., stating that preservation does not affect shape or size when in fact it does). Thus, we assessed significance with and without the sequential Bonferroni method.

## 3 Results

**FIGURE 2:**
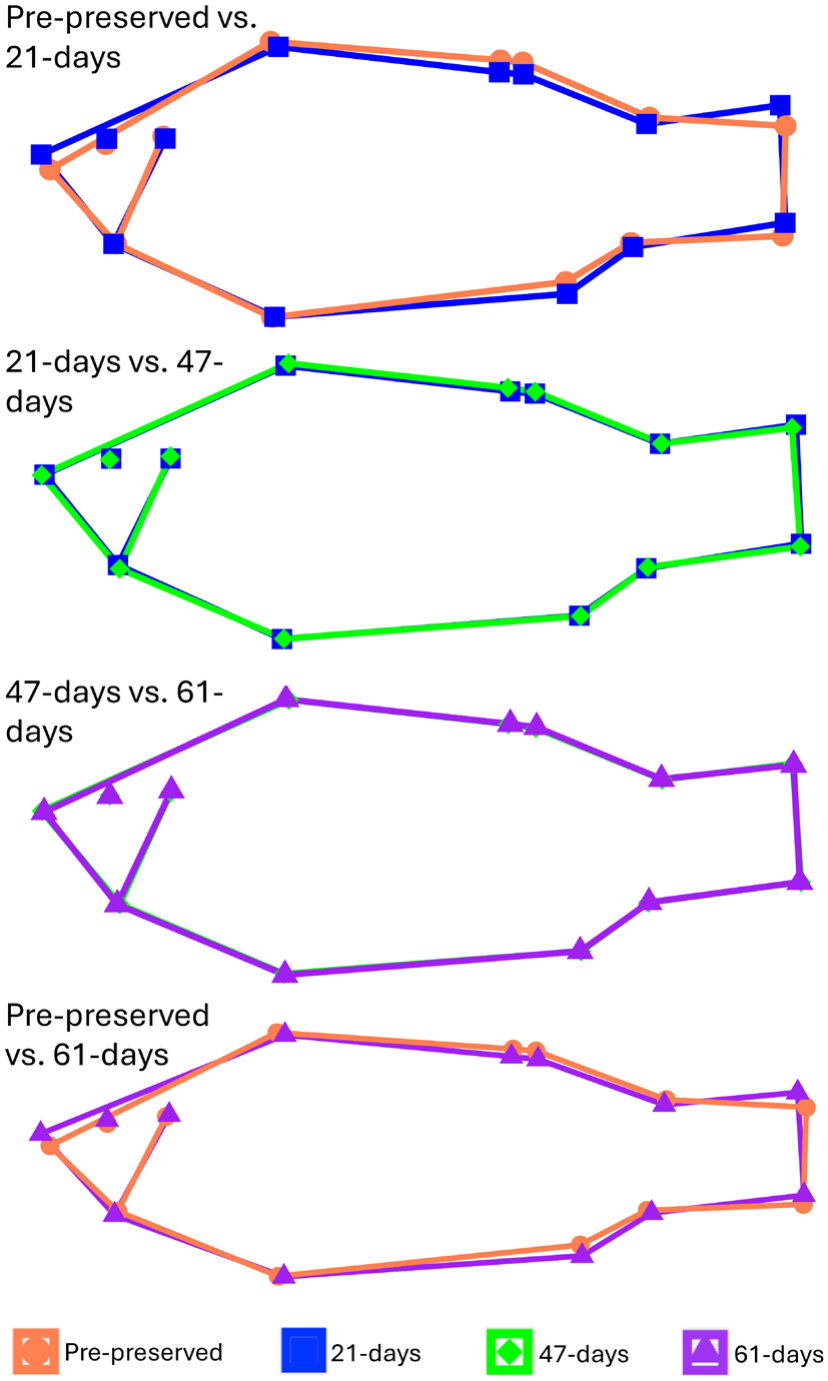
Wireframe plots of size‐independent shape between time periods. Each shape represents the average configuration of landmarks for a given time period after accounting for allometry.

Prior to fixation and preservation, the mean (±1 SD) size of Yellow Perch was 283.9 ± 32 mm TL (range: 202–362 mm TL), 301.1 ± 109 g wet mass (range: 125–711 g), and 298.3 ± 42.6 centroid size (range: 213.65–404.9). The shape of preserved Yellow Perch changed over time. Repeated‐measures tests identified significant differences in body shape for pre‐preservation versus 21 days, 21 versus 47 days, and pre‐preservation versus 61 days, but not for 47 versus 61 days (Table 1; Figure 2). The interpretation of significance did not change when we used the sequential Bonferroni method (Table 1). The most prominent changes were observed when comparing pre‐preservation vs. 21 days, with subsequent time periods showing comparatively less variation (as measured by the Euclidean distance between the means of each time period). Wireframe plots showed that dorsal bending and expansion of the head and caudal region occurred for the comparison of pre‐preservation vs. 21 days, with more fine‐scale differences occurring for the comparison of 21 vs. 47 days (Figure 2). Although not part of our statistical analyses, lateral bending was observed in 26 out of 42 specimens between pre‐preserved and 61 days.

**TABLE 1:** Statistical results for differences in shape between time periods. *p*‐values deemed significant after applying the sequential Bonferroni method are indicated with an “*”.

Comparison	Hotelling's *T* ^2^	Euclidean distance	*F*_1,4_	*p*
Pre‐preserved vs. 21 days	810.20	0.033	17.964	< 0.001*
21 vs. 47 days	183.55	0.010	4.070	0.001*
47 vs. 61 days	55.98	0.006	1.241	0.315
Pre‐preserved vs. 61 days	875.48	0.031	19.412	< 0.001*

*Note:* To apply the sequential Bonferroni method, rank *p*‐values (*p*_1_ < *p*_2_ < *p*_3_ < *p*_4_, where *i* = 1 to *k* and *k* = 4) and evaluate significance based on *p*_*i*_ ≤ *α*/[1 + *k*—*i*] (Peres‐Neto 1999). Setting *α* = 0.05, significant values for *i* = 1 … 4 are 0.013, 0.017, 0.025, and 0.050, respectively.

In addition to shape changes, we also observed changes in size (i.e., mass, total length, and centroid size) over time; although few comparisons remained significant once we applied the sequential Bonferroni method. Paired *t*‐tests identified a significant change in mass for pre‐preserved vs. 21 days and pre‐preserved vs. 61 days, but those were no longer significant when applying the sequential Bonferroni method (Table 2). Additionally, in the comparison of 47 days vs. 61 days, we observed a significant decrease in centroid size and a significant increase in total length; although only total length remained significant once we applied the sequential Bonferroni method (Table 2). Regardless, the magnitude of the differences in size was small (Table 2), especially for total length (~2 mm) and mass (~1 g).

**TABLE 2:** Statistical results from paired *t*‐tests for comparisons of centroid size, total length (mm), and mass (g) between time periods. Mass was transformed to meet the assumption of normality. A positive mean difference indicates a decrease in the response variable from time *j* to *j* + 1; whereas a negative value indicates an increase. *p*‐values deemed significant after applying the sequential Bonferroni method are indicated with an “*” (Table 1 for adjusted significance criteria).

Response variable	Comparison	*t*_41_	Mean difference	Confidence interval	*p*
Centroid size	Pre‐preserved vs. 21 days	1.360	4.339	−2.106, 10.78	0.181
	21 vs. 47 days	1.571	7.482	−2.137, 17.10	0.124
	47 vs. 61 days	−2.561	−15.051	−26.92, −3.182	0.014
	Pre‐preserved vs. 61 days	−0.663	−3.231	−13.07, 6.612	0.511
Total length	Pre‐preserved vs. 21 days	0.711	2.619	−4.822, 10.06	0.481
	21 vs. 47 days	−0.575	−1.905	−8.599, 4.789	0.569
	47 vs. 61 days	2.951	1.952	0.616, 3.287	0.005*
	Pre‐preserved vs. 61 days	1.332	2.667	−1.376, 6.710	0.190
ln(Mass)	Pre‐preserved vs. 21 days	−2.332	−0.020	−0.037, −0.003	0.025
	21 vs. 47 days	−0.766	−0.003	−0.011, 0.005	0.448
	47 vs. 61 days	1.949	0.005	−0.000, 0.009	0.058
	Pre‐preserved vs. 61 days	−2.284	−0.018	−0.035, −0.002	0.026

## 4 Discussion

We found that formalin fixation and preservation in ethanol can alter the body shape and size of adult Yellow Perch over a 2‐month period, often supporting our original hypotheses. However, the magnitude and timing of these changes varied, sometimes in unexpected ways. For instance, shape and possibly mass showed an initial effect (i.e., pre‐preserved vs. 21 days) but then minimal change thereafter, whereas we only detected differences in total length and possibly centroid size further along in preservation (i.e., 47 vs. 61 days). We observed a small (1 g or 0.3% of mean body mass) initial (pre‐preserved vs. 21 days) decrease in mass, which was consistent with our original hypotheses and other studies showing size decreases early in the preservation process (Lee et al. 2012; Shields and Carlson 1996). In contrast, we observed a small (2 mm or 0.7% of mean TL) decrease in length and a moderate (5.1%) increase in centroid size toward the end of the preservation process. Regardless, the changes in total length and mass represented less than 1% change from the average. In addition to changes caused by the preservation process, measurement error (Bunch et al. 2013; Fruciano 2016) and Type I statistical error (Peres‐Neto 1999) provide alternative explanations. While these differences appear small, Engel (1974) found that even slight differences in length (0.7%–2.1%) and mass (1.7%–5.0%) measurements can greatly influence calculations of condition factor. Assuming that the changes in centroid size and total length were caused by the preservation process, it represents an interesting occurrence where size changes after more than a month of preservation. Additionally, the changes in total length were not similar to those found in many other studies. Across species, preserved specimens commonly exhibit a reduction in length over time (Leslie and Moore 1986; Shields and Carlson 1996; Cunningham et al. 2000; Lee et al. 2012). However, in our study, we did not observe a significant decline in total length over time. Instead, we observed no significant difference in total length between pre‐preserved and 61 days, which contradicted our original hypothesis.

Preservation induced changes in body shape are commonly observed in other fishes (Sotola et al. 2019; Fruciano et al. 2020; Lorencen et al. 2021). In the present study, many specimens became dorsally and ventrally bent after 21 days in ethanol, which is a common effect of preservation (Carpenter 1996; Cavalcanti et al. 1999; Fruciano et al. 2020). Whether specimens continued to become more bent over time is unknown, as we attempted to manually straighten specimens when pinning them for photos because our focus was on actual shape changes (i.e., shrinkage or enlargement of certain body parts vs. bending). While both dorsal and ventral bending were observed, dorsal bending visually appeared to be of greater magnitude. There are multiple methods for removing the bending effect, such as Burnaby's procedure (Burnaby 1966; Valentin et al. 2008), using the “unbend specimens” feature in tpsUtil software (Rohlf 2018), or simply excluding “bent” specimens from statistical analyses. However, the best approach is that future studies should try to orient specimens in positions (e.g., straight with fins flared) during the preservation process that are least likely to result in bending and require manipulation of specimens for photographing. We also visually observed lateral bending (that could not be quantified based on the orientation of our photos), which was present in 62% of specimens after 61 days of preservation. In addition to preservation induced bending, changes including expansion of the head and caudal region were observed and were of greatest magnitude initially (pre‐preserved vs. 21 days) in the preservation process. Shape comparison in the mid time period (21 vs. 47 days) revealed an expansion of the body and slight ventral bending of the caudal region toward the pre‐preservation state. These findings suggest that in addition to bending, preservation can alter certain segments of the body more than others.

Previous work showed that the effects of preservation can be species specific (Sotola et al. 2019). Our study showed that adult Yellow Perch were affected by preservation. The effects of preservation on Yellow Perch have been shown to vary by size and the method of preservation (Stobo 1972; Engel 1974; Fisher et al. 1998; Paradis et al. 2007). Paradis et al. (2007) observed a minor (1.2%) reduction in Yellow Perch lengths, aligning closely with our findings. However, in contrast to our observations, Paradis et al. (2007) also revealed a substantial 26% decrease in mass. The disparity could be attributed to preservation specifics, such as the absence of formalin fixation or variations in the ethanol concentration. Another factor could be the size of specimens used in the study. The response to preservation can be strongly influenced by the stage and size of preserved specimens, which was demonstrated by the distinct effects observed on larval and juvenile Yellow Perch (Paradis et al. 2007). Our study showed the effects of a standard preservation technique (formalin fixation and ethanol preservation; Hubbs and Lagler 2004) on the shape and size of adult Yellow Perch. Based on these findings, we recommend that researchers: (i) use fresh fish whenever possible, (ii) if fresh fish are not available or their use is impractical, then use the conservative approach of measuring/photographing specimens that have been preserved for the same amount of time, and (iii) if only archival specimens are available, then select specimens that minimize the difference in duration that specimens were preserved even though the effects of long‐term (e.g., > 10 years) preservation remain unresolved. Finally, permanently archiving photographs of fish, with detailed information regarding how fish were handled (e.g., fresh or duration and details of preservation) prior to photographing, should provide valuable information for future analyses of fish morphology while alleviating the unresolved challenge of how the shape of preserved specimens changes over decades.

## Author Contributions

**Tyler J. Hoyt:** conceptualization (equal), data curation (lead), formal analysis (lead), funding acquisition (supporting), investigation (equal), methodology (equal), project administration (equal), resources (supporting), software (lead), supervision (supporting), validation (supporting), visualization (lead), writing – original draft (lead), writing – review and editing (equal). **Carl R. Ruetz III:** conceptualization (equal), formal analysis (supporting), funding acquisition (lead), investigation (equal), methodology (equal), project administration (supporting), resources (lead), supervision (lead), validation (lead), writing – original draft (supporting), writing – review and editing (equal).

## Conflicts of Interest

The authors declare no conflicts of interest.

## Data Availability

Size measurements and .tps files available in the Dryad digital repository at https://doi.org/10.5061/dryad.cfxpnvxhs.
